# Relationships Among the EmPHasis-10 Questionnaire, the Simplified Four-Strata Risk Assessment Tool, and Echocardiographic Parameters in Patients with Precapillary Pulmonary Hypertension

**DOI:** 10.3390/jcm13226782

**Published:** 2024-11-11

**Authors:** Andreea Varga, Liviu Cristescu, Dragos-Gabriel Iancu, Robert-Adrian Dumbrava, Diana-Andreea Moldovan, Florin Stoica, Stefania Raluca Fodor, Claudiu Neagu, Radu Adrian Suteu, Ioan Tilea

**Affiliations:** 1 Faculty of Medicine in English, George Emil Palade University of Medicine, Pharmacy, Science and Technology of Targu Mures, 540142 Targu Mures, Romania; andreea.varga@umfst.ro (A.V.); diana.moldovan@umfst.ro (D.-A.M.); 2 Department of Internal Medicine II-Cardiology, Emergency Clinical County Hospital, 540042 Targu Mures, Romania; dragos-gabriel.iancu@umfst.ro (D.-G.I.); robert-adrian.dumbrava@umfst.ro (R.-A.D.); stoica.florin.23@stud.umfst.ro (F.S.); ioan.tilea@umfst.ro (I.T.); 3 Doctoral School, George Emil Palade University of Medicine, Pharmacy, Science and Technology of Targu Mures, 540142 Targu Mures, Romania; claudiu.neagu@umfst.ro; 4 Faculty of Medicine, George Emil Palade University of Medicine, Pharmacy, Science and Technology of Targu Mures, 540142 Targu Mures, Romania; raluca.fodor@umfst.ro; 5 Department of Cardiology I, The Emergency Institute for Cardiovascular Diseases and Transplantation, 540136 Targu Mures, Romania; radu.suteu@umfst.ro; 6 Department of Anesthesiology and Intensive Care, Emergency Clinical County Hospital, 540042 Targu Mures, Romania; 7 Department of Public Health, Emergency Clinical County Hospital, 540136 Targu Mures, Romania

**Keywords:** pulmonary hypertension, patient perspective, EmPHasis-10 questionnaire, risk stratification, four-strata risk assessment, cardiac ultrasound

## Abstract

**Background/Objectives**: Pulmonary arterial hypertension (PAH) and chronic thromboembolic pulmonary hypertension (CTEPH) are complex diseases that require precise diagnosis and management. The ESC risk score has been used in both conditions. We assessed the relationship between the EmPHasis-10 questionnaire (patient subjective evaluation) and objective assessment using endorsed tools (simplified four-strata risk assessment and right ventricular imaging by transthoracic echocardiography). **Methods**: The present study retrospectively extracted data from 40 adult patients (27 PAH and 13 CTEPH cases) diagnosed in a single center in Romania. The EmPHasis-10 questionnaire and the four-strata risk assessment (FSRA) tool were applied to each patient. Mean pulmonary artery pressure (mPAP), tricuspid annular plane systolic excursion (TAPSE), TAPSE/systolic pulmonary artery pressure (TAPSE/sPAP) ratio, and right ventricular outflow tract acceleration time (RVOT-AT) were assessed. **Results**: A significant correlation was observed between the EmPHasis-10 scores and the FSRA tool, the WHO functional class, and the 6 min walking distance. Emphasis-10 score did not correlate with any of the echocardiographic parameters. The FSRA tool showed a moderate positive correlation with mPAP (r = 0.42, *p* = 0.01) and a negative correlation with TAPSE (r = −0.46, *p* = 0.003); additionally, across the entire cohort, it was moderately negatively correlated with both RVOT-AT (r = −0.42, *p* = 0.01) and TAPSE/sPAP ratio (r = −0.43, *p* = 0.005). **Conclusions**: Our study evidenced the alignment between EmPHasis-10 scores and prognostic risk score, with poorer health-related quality of life corresponding to higher FSRA. The EmPHasis-10 questionnaire proves to be a valuable, easy-to-use instrument, offering meaningful insights into patients’ health-related quality of life, underscoring its utility in enhancing comprehensive patient assessment and management.

## 1. Introduction

Pulmonary hypertension (PH) is a significant global health issue with an estimated worldwide prevalence of 1%. The European Society of Cardiology/European Respiratory Society (ESC/ERS) 2022 Guidelines for the diagnosis and treatment of PH focus on risk stratification as a basis for disease management [1].

In recent decades, significant advances have been made in the treatment of PH patients. However, PH patients experience various nonspecific symptoms that affect their health-related quality of life (HRQoL) [2]. The ESC/ERS 2022 Guidelines highlight that patient-reported outcomes are heavily underused. The EmPHasis-10 questionnaire is a validated, accessible, and well-suited instrument used in daily practice to evaluate HRQoL in PH patients by exploring patients’ perspectives. The questionnaire comprises 10 components, each evaluated on a scale from 0 to 5, designed to assess several key factors, including dyspnea, fatigue, low energy levels, social limitations, and the perception of being a burden to family and friends [3].

The EmPHasis-10 questionnaire score has potential not only as a tool for assessing quality of life but also offering a quantifiable metric that correlates strongly with patient outcomes. The prognostic utility of the EmPHasis-10 questionnaire score among PH patients revealed that each 10-unit increase in the abovementioned score was associated with a 1.53-fold increase in the mortality hazard of the overall PH cohort, underlining the score’s effectiveness as an independent predictor of mortality [4].

The EmPHasis-10 questionnaire’s validity supports its use as a reliable, rapid tool for risk stratification, significantly predicting the likelihood of achieving a low-risk profile at 12-month follow-up [5,6].

In precapillary PH, EmPHasis-10 questionnaire scores are significantly correlated with both neurohormonal indices and hemodynamic parameters (mean pulmonary artery pressure and pulmonary vascular resistance), reflecting the impact of cardiac performance on the patient-perceived health status [7].

Researchers have confirmed that changes in cardiac MRI metrics correlate with improvements in patient-reported outcomes quantified by the EmPHasis-10 questionnaire, validating the clinical significance of minimal metric differences for PAH therapies [8].

Known prognostic factors are conventionally used to estimate the mortality risk of PH patients with a comprehensive three-strata tool that estimates the 1-year mortality rates [1]. Two European registries (Compera 2.0 and the French PAH registry) analyzed a new four-strata model risk assessment tool (FSRA) and validated it in PAH patients [9,10].

The 6 min walking distance (6MWD) is related to age, height, weight, and various comorbidities across the PH subgroups [1,11,12]. It is a simple, repeatable, economical, and accessible test used to assess the clinical status, prognosis, and treatment follow-up in PH cases. A 6MWD greater than 440 m indicates a low-risk profile in PH patients [1,13,14,15].

N-terminal pro-B-type natriuretic peptide (NT-proBNP) is a key biomarker for diagnosing and managing pulmonary hypertension by indicating right ventricular dysfunction. Integrated into international guidelines over the past two decades, it aids in assessing disease severity, guiding treatment, and predicting outcomes, complementing other diagnostic methods [16]. In PAH patients, NT-proBNP levels correlate better with prognosis, although selected groups of patients require higher cut-off value concentrations [17,18,19].

The FSRA effectively predicted the 10-year survival in connective-tissue-disease-associated PAH patients, highlighting improvements in treatment and survival outcomes, especially after 2015, due to an increased use of targeted combination therapies [20]. In a specific subset of patients with combined pre- and postcapillary PH, the application of the FSRA effectively stratified the survival risks, emphasizing its importance in the clinical management of this PH subgroup [21].

According to current studies, the presence of different comorbid diseases in PAH patients has a negative impact on outcomes [22,23,24,25].

To date, there is still no established risk assessment with the aim of guiding treatment decisions specifically for medically managed CTEPH patients [25]. However, the existing literature argues that the application of the FSRA tool can effectively stratify risk among medically managed CTEPH patients, even though it was originally developed for PAH patients. This stratification aligns with the outcomes reported in existing studies [26,27,28].

PH gradually leads to right ventricular overload and progressive dysfunction. The 2022 ESC/ERS Guidelines outline a number of PH echocardiographic findings, that provide accurate information about heart status and function, and they can be used to estimate certain hemodynamic parameters using indirect formulas. Right heart catheterization is necessary to confirm a diagnosis of PH, as echocardiography alone does not provide sufficiently precise hemodynamic data [1]. The need for further research into the role of echocardiographic assessment of the right ventricle for risk stratification in PH, especially in PAH and CTEPH, remains.

This study aimed to assess the hypothesis that noninvasive accessible tools (the EmPHasis-10 questionnaire, the FSRA, or a combination of both) are correlated with echocardiographic parameters and can be utilized in the assessment of patients with PAH and CTEPH, as these two phenotypes of PH are amenable to targeted medical, surgical, and interventional therapies. This hypothesis was tested using data from a single PH center in Romania.

## 2. Materials and Methods

This study is a retrospective, descriptive, noninterventional analysis of a single-center PH registry from the Pulmonary Hypertension Center at the County Emergency Clinical Hospital of Targu Mures, Romania. The local registry included 65 adult patients diagnosed with PAH or CTEPH in accordance with the 2015 and subsequently updated 2022 ESC/ERS Guidelines.

From October 2022 to May 2023, aggregated and anonymized patient data were collected from both electronic and paper-based medical records. Inclusion criteria, irrespective of PH clinical classification, mandated the collection of the following variables: age, sex, body mass index (BMI), environmental factors, educational level, employment status (employed, disabled, or retired), World Health Organization functional class (WHO-FC), 6MWD, NT-proBNP levels, echocardiographic parameters, right heart catheterization data, and responses to the EmPHasis-10 questionnaire.

Upon a data review, 25 patients were excluded due to a lack of the requested information or loss to follow-up. For the final analysis, 40 cases were eligible.

According to their clinical classification, the enrolled patients were divided into two groups: PAH group and CTEPH group.

PAH group comprised 27 patients, stratified according to distinct underlying etiologies. This cohort included 7 patients with idiopathic PAH (IPAH), 1 patient with PAH associated with connective tissue disease (PAH-CTD), 1 patient with PAH associated with portal hypertension (PoPH), and 18 patients with PAH associated with congenital heart disease (PAH-CHD).

Within the studied period, the medical regimen for the PAH patients included approved drugs (PDE5 inhibitors—sildenafil; endothelin receptor antagonists—bosentan and macitentan) administered either as mono or double therapy.

At the time of the study, other medications targeting the prostacyclin pathway—such as selexipag, a selective prostacyclin receptor agonist, and treprostinil, a synthetic prostacyclin analog—were not yet available for clinical use.

For patients with CTEPH, none had undergone surgery or balloon pulmonary angioplasty at the time of enrollment. Riociguat was the standard therapy in use for these patients in the entire study period.

All procedures were carried out according to local standard practice. BMI was estimated using Mosteller’s formula. Environment and employment status were also included in the entire cohort description. The level of education was quantified using the International Standard Classification of Education ISCED 2011 [29].

The 6MWD was performed as stated in the American Thoracic Society Statement guidelines for the six-minute walk test in a 30 m corridor marked at 3 m [15].

The values for NT-proBNP were obtained using a Nano-Checker™ 710 reader (Nano-Ditech Corporation, Cranbury, NJ, USA) system. The same assay kits were used to analyze each patient’s blood sample.

Transthoracic echocardiography was performed using a GE Vivid™ E9 System (GE Vingmed Ultrasound AS, Horten, Norway). From all accepted echocardiographic parameters endorsed by current guidelines for the diagnosis, management, and follow-up of PH patients, eight were selected for use in our study [1].

The systolic peak velocity tricuspid valve (peak TRV), tricuspid annular plane systolic excursion (TAPSE), right ventricular outflow tract acceleration time (RVOT-AT), tricuspid regurgitation pressure gradient, and inferior vena cava (IVC) diameter and collapse were assessed for each patient. Systolic pulmonary artery pressure (sPAP) was computed by summing the tricuspid regurgitation (TR) pressure gradient assessed in apical four-chamber view with the estimated right atrial pressure, and the result was used to determine the TAPSE/sPAP ratio. Mean pulmonary artery pressure (mPAP) was estimated using the following formula: mPAP = 90 − (0.62 × RVOT-AT) [30]. In the studied cohort, these parameters were only partially obtained for three patients with complex congenital heart disease due to their individual anatomical situations.

The hemodynamic parameters measured via right heart catheterization, namely the mean pulmonary artery pressure (mPAP), pulmonary artery wedge pressure (PAWP), pulmonary vascular resistance (PVR), cardiac index (CI), and cardiac output (CO), are detailed in Table 1.

The Romanian version of the EmPHasis-10 questionnaire was provided to each patient on the basis of self-assessment evaluation.

In our study, we also assessed the mortality rates (based on the four-strata risk assessment) and compared them with those reported by the 2022 ESC/ERS Guidelines [1]. Particularly, for the PAH group, the mortality rates were assessed based on the Emphasis-10 questionnaire scores divided into three intervals using the cut-off values from a model proposed by Lewis et al.: 0–16, 17–33, and 34–50 points [31].

The collected data encompassed the entire population and were processed using Microsoft 365^®^ Excel^®^ (Microsoft Corporation, Redmond, WA, USA). Analyses were performed with GraphPad Prism version 9.3.0 for Windows (GraphPad Software, San Diego, CA, USA). Categorical data are reported as frequencies (percentages), while parametric continuous data are expressed as mean values ± the standard deviation (SD). Nonparametric continuous data are presented as medians (minimum–maximum). Statistical significance was determined at a *p*-value of ≤0.05. The Kolmogorov–Smirnov test was employed to assess the normality of continuous data. Correlations were evaluated using Pearson’s r for parametric variables and Spearman’s r for nonparametric variables. Differences between groups were assessed using the Student’s *t*-test for parametric variables and the Mann–Whitney U test for nonparametric variables.

## 3. Results

The present study included data from 40 adult patients, with their baseline characteristics summarized in Table 1. The mean age of the entire population was 53.18 ± 18.56 years, and 23 patients (57.5%) were female. The entire cohort included 27 (67.5%) PAH patients and 13 CTEPH patients (32.5%).

Among patients with PAH-CHD, comprising 66.66% of the PAH group (n = 27), the mean age was 42.11 ± 18.18 years. Of these patients, 9 (33.33%) were female, 10 (37.03%) resided in urban areas, and 11 (40.74%) had attained International Standard Classification of Education level 3 education. Seven patients (25.92%) were classified as WHO functional class II and nine (33.33%) achieved a 6MWD exceeding 440 m. Additionally, nine patients exhibited NT-proBNP levels higher than 300 pg/mL.

Due to the distinct etiological and pathophysiological mechanisms of both groups, overall and group-related data were analyzed to assess differences. PAH patients were significantly younger than CTEPH patients (*p* = 0.028).

**Figure 1:**
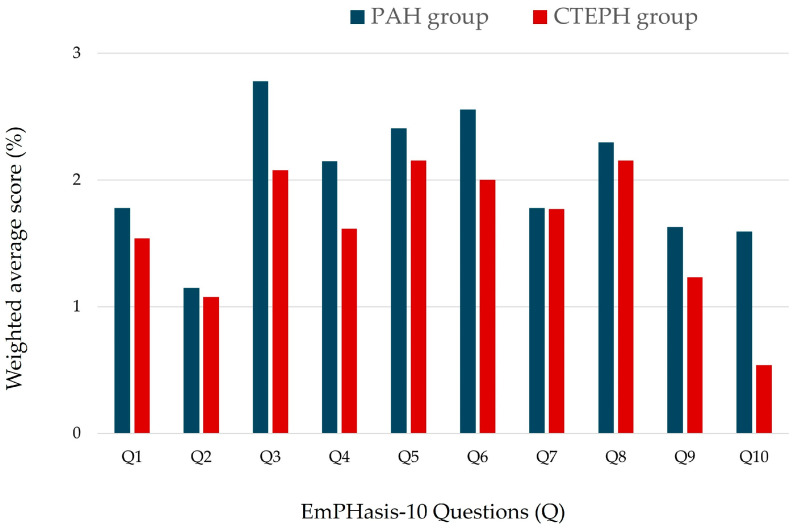
The weighted average score for PAH group and CTEPH group.

The EmPHasis-10 questionnaire was applied to all cases, and the highest weighted question was item number 3 (“I do not/always need to rest during the day”; see Figure 1). The average response value for the entire cohort was 18.83 ± 12.71 points (data are presented in Table 2).

We hypothesized that a higher level of education might influence the application of the EmPHasis-10 questionnaire. However, when analyzed using the ISCED 2011 classification, no significant differences in education levels were identified between PAH group and CTEPH group.

Furthermore, no correlation was identified between level of education and the EmPHasis-10 questionnaire score, either for the entire cohort (r = 0.1, *p* = 0.542) or when analyzed separately by groups (PAH group: r = 0.03, *p* = 0.873; CTEPH group: r = 0.17, *p* = 0.575).

A point-biserial correlation between the patient’s environment (rural/urban) and EmPHasis-10 questionnaire score (rpb = −0.1, *p* = 0.537) was not identified for the entire cohort, nor for the PAH group or CTEPH group, meaning that the environment did not influence the questionnaire score.

Applying the FSRA tool, a mean of 2.25 ± 0.98 points was obtained for the entire cohort. Additionally, we present the stratification of FSRA scores by WHO functional class (see Table 3).

**Figure 2:**
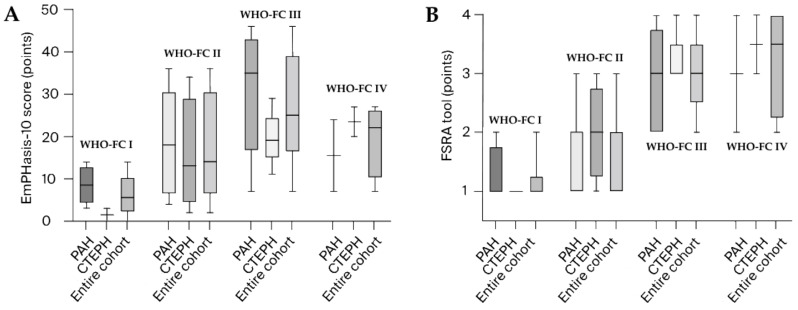
EmPHasis-10 score (**A**) and FSRA tool (**B**) distribution according to WHO-FC subgroups, framed on PAH patients, CTEPH patients, and the entire cohort.

The distribution of the FSRA tool points and the EmPHasis-10 questionnaire scores stratified by WHO-FC (see Figure 2), compared between the PAH group and CTEPH group, revealed no significant differences (data are depicted in Table 2 and Table 3, respectively).

**Figure 3:**
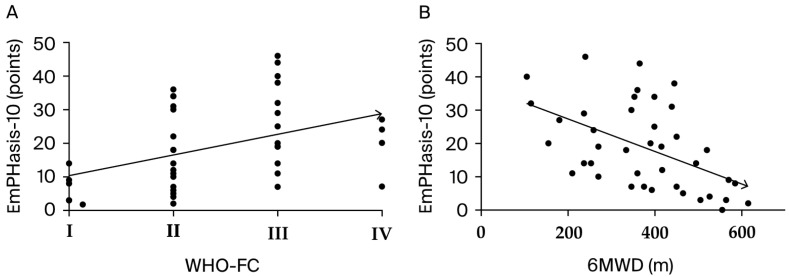
Linear regression performed for the entire cohort between the WHO-FC and the EmPHasis-10 questionnaire score (**A**) and the 6MWD and the EmPHasis-10 questionnaire score, respectively (**B**).

For the entire cohort, there was a significant positive correlation between the WHO-FC and EmPHasis-10 questionnaire scores (r^2^ = 0.176, *p* = 0.007). In contrast, the 6MWD (366.4 ± 138.3 m) and EmPHasis-10 questionnaire scores showed a significant negative correlation (r^2^ = 0.257, *p* < 0.001) (see Figure 3).

**Figure 4:**
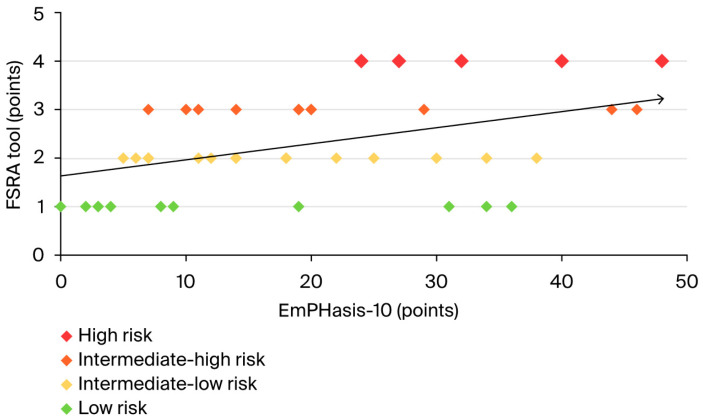
Pearson’s correlation for the entire cohort between the EmPHasis-10 questionnaire and the FSRA tool.

Our study demonstrated a moderate, significant positive correlation between the EmPHasis-10 questionnaire score and the FSRA tool (r = 0.38, *p* = 0.014, Pearson’s correlation) across the entire population (see Figure 4). This indicates that the questionnaire scores aligned with the FSRA, with higher FSRA risk scores corresponding to a worse subjective assessment of the patient’s status.

The serum levels of natriuretic peptides (1914.88 ± 2569.28 pg/mL) were not significantly correlated with the EmPHasis-10 questionnaire score (r^2^ = 0.02, *p* = 0.816). This could be explained by the fact that NT-proBNP values can be affected by the presence of comorbidities (particularly age and eGFR values) or atrial fibrillation, as these factors are responsible for increasing natriuretic peptide levels.

Echocardiographic parameters were assessed and compared between the PAH group and CTEPH group; data are presented in Table 4.

The results derived from Spearman’s correlation between the EmPHasis-10 questionnaire score and the echocardiographic parameters for the entire cohort did not outline a significant correlation (see Table 5). However, for the FSRA tool, there was a moderate positive correlation for mPAP (r = 0.42, *p* = 0.01), a moderate negative correlation with TAPSE (r = −0.46, *p* = 0.003), a moderate negative correlation for the entire cohort with TAPSE/sPAP ratio (r = −0.43, *p* = 0.005), and a moderate negative correlation with RV-AOT (r = −0.42, *p* = 0.01).

Subsequently, in the PAH group, the FSRA tool was directly correlated with mPAP (+0.57, *p* = 0.004) and inversely correlated with RVOT-AT (−0.47, *p* = 0.013) and TAPSE (−0.527, *p* = 0.006). An inverse correlation between the FSRA and TAPSE was also identified in the CTEPH patients (−0.56, *p* = 0.046); however, the echocardiographic parameters (independent variables) were not found to be predictors of the FSRA score (dependent variable) after the ANOVA test assessment, regardless of the group phenotype (p _PAH group_ = 0.152, r^2^_PAH group_ = 0.556; p _CTEPH group_ = 0.191, r^2^_CTEPH group_ = 0.817).

**Figure 5:**
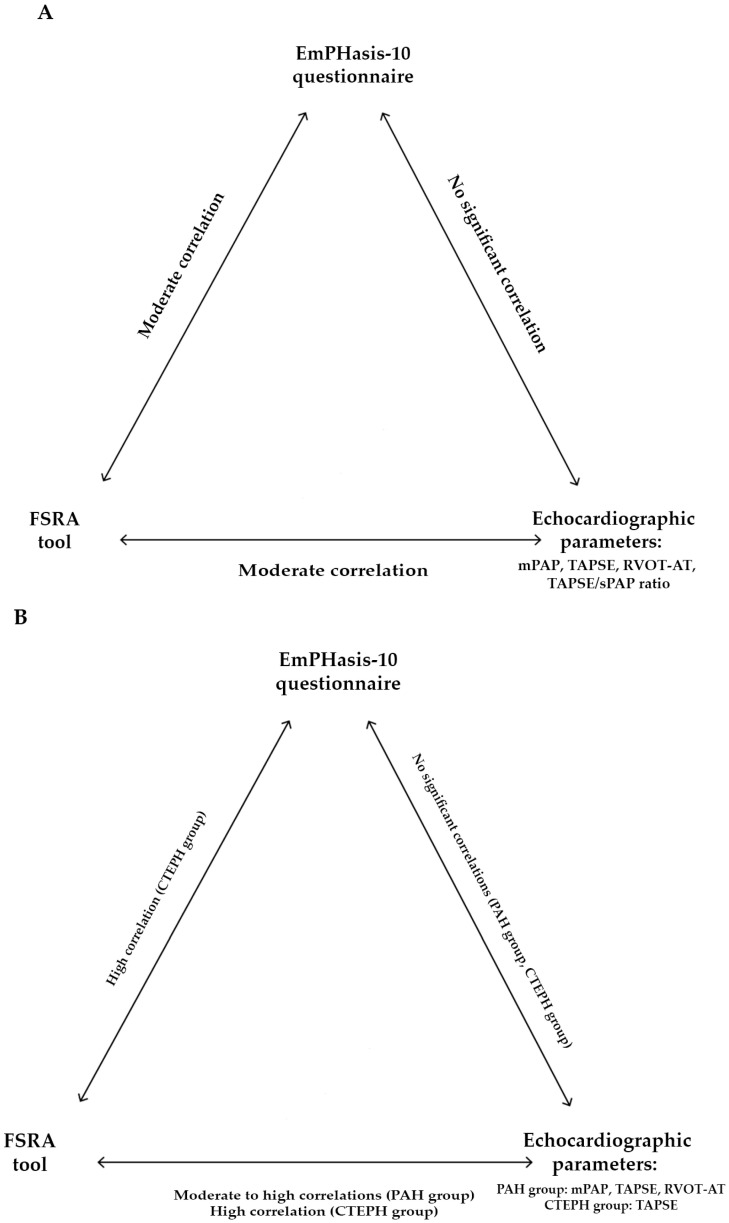
The key findings from the current study are illustrated for the entire cohort (**A**), as well as for the patients with PAH and for the CTEPH patients (**B**). FSRA, four-strata risk assessment tool; mPAP, mean pulmonary artery pressure; RVOT-AT, right ventricular outflow tract acceleration time; sPAP, systolic pulmonary arterial pressure; TAPSE, tricuspid annular plane systolic excursion.

In examining patient perspectives, the risk assessment, and echocardiographic parameters, our investigation aimed to pinpoint any robust predictors within the echocardiographic parameters for the EmPHasis-10 questionnaire or the FSRA tool (see Figure 5).

Over the course of follow-up in this study, four patients died (two PAH and two CTEPH). Twelve-month mortality utilizing the FSRA tool corresponded to the tool estimates (Table 6).

For the PAH group, we compared one-year mortality rates to the data reported by Lewis et al., based on mortality stratified by EmPHasis-10 questionnaire scores [31]. In our research, one-year mortality rates identified one death (7.69%) in the 0–16 EmPHasis-10 questionnaire score range and one death in the 17–33 EmPHasis-10 questionnaire score range.

## 4. Discussion

Numerous studies involving the EmPHasis-10 questionnaire or FSRA tool have been conducted on PH subjects. This is the first study to assess the relationship between health-related QoL scores, risk assessment tools, and echocardiographic parameters in Romanian PAH and CTEPH patients. Our research confirms that the EmPHasis-10 questionnaire is a valuable tool for both group 1 and group 4 PH patients in accordance with the 2022 ESC/ERS Guidelines and should be utilized for Romanian PH patients receiving targeted therapies. Furthermore, we found a correlation between the WHO-FC and the EmPHasis-10 questionnaire as well as between the 6MWD and the EmPHasis-10 questionnaire. Compared with our study, where CTEPH patients had an average of 16.15 ± 10.52 points for the EmPHasis-10 questionnaire score, Takeyasu et al. reported a score of 19.4 ± 10.6 points in a Japanese cohort, which correlated with worsening WHO-FC [32]. A possible explanation for our lower mean values could be the difference in the proper comprehension of the EmPHasis-10 questionnaire, as the answers are subjectively dependent on the patient’s self-perception and self-grading of the disease.

A study involving 565 patients from the Pulmonary Hypertension Association Registry (United Kingdom) concluded that the 6MWD and B-type natriuretic peptide (BNP)/N-terminal proBNP levels were also significantly associated with EmPHasis-10 questionnaire scores both at baseline and over time [33]. In contrast, our study observed fluctuations in NT-proBNP levels, likely due to the wide range of comorbidities among patients and the small cohort size characteristic of single-center studies. Nonetheless, EmPHasis-10 questionnaire scores did not correlate with the NT-proBNP values, possibly due to the various factors influencing this biomarker’s serum concentrations.

In our CTEPH group, the EmPHasis-10 questionnaire scores presented a moderate correlation with the FSRA score. An explanation for this could be that the best survival prognosis among all PH groups defined by the guidelines is known to be for CTEPH patients.

In terms of the relationship between the 12-month mortality and the four-strata risk assessment, our research obtained comparable mortality rates to those presented in the current guidelines. However, it is important to acknowledge that this is a relatively small cohort, with a limited number of patients in the high-risk group. As a result, there is a possibility that causality factors may have a statistical distribution effect on the mortality analysis.

Lewis et al. reported moderate correlations between baseline EmPHasis-10 questionnaire and WHO-FC and 6MWD and weak correlations with right heart catheterization parameters, namely mPAP, cardiac index, and PVR in idiopathic PAH, associated with drugs, heritable PAH, and PAH-CTD [31].

In applying their model to our PAH group, mortality was observed in patients with EmPHasis-10 scores ranging from 0 to 16 and 17 to 33 points, but these results should be interpreted very carefully because we are reporting a relatively small cohort with a limited number of patients in the high-risk group. The limited number of patients and even the geographical area could explain the observed differences. The results could also be influenced by the patients’ levels of understanding and self-grading of the EmPHasis-10 questionnaire.

Hendriks et al. [34] assessed echocardiographic parameters, among multiple variables, against the EmPHasis-10 and CAMPHOR questionnaires’ HRQoL at baseline, six months, and twelve months. The questionnaire maintained its correlation with the 6MWD at baseline and during follow-up; however, poor correlations with NT-proBNP levels were obtained throughout the study. The authors noticed weak correlations between the echocardiographic data at baseline, the HRQoL domain, and TAPSE. Notably, in our study, four of eight echocardiographic parameters (mPAP, TAPSE, TAPSE/sPAP ratio, and RVOT-AT) demonstrated significant correlations with the FSRA tool in the full cohort.

While our results are not directly comparable to studies targeting patients with pulmonary arterial hypertension and chronic thromboembolic pulmonary hypertension, TAPSE and the TAPSE/sPAP ratio—which reflects right ventricular–pulmonary arterial coupling—are commonly used as conventional echocardiographic indices to evaluate right ventricular dysfunction [35]. However, in the setting of severe tricuspid regurgitation (TR), their utility is limited as a result of changes in the volume and pressure of the right ventricle. Elevated right ventricular preload or volume overload lead to overestimation, secondary to the increasing regurgitant blood volume into the right atrium, which enhances the TAPSE even without any increase in the RV myocardial contractility. Studies assessing the prognostic significance of right ventricular dysfunction have reported controversial results, likely due to the aforementioned limitations of these parameters [36,37,38,39].

Minhas et al. evaluated the EmPHasis-10 questionnaire’s role in HRQoL of the patients with IPAH and CTEPH undergoing medical management, showing that EmPHasis-10 scores were not different over time in these two groups [40]. Building on their approach of comparing different pulmonary hypertension subgroups, our study identified that EmPHasis-10 scores did not differ between PAH and CTEPH patients that were stable and pharmacologically managed, further underscoring the utility of this questionnaire in diverse PH populations and highlighting the need for further research in this area.

While the present research demonstrated essential associations between EmPHasis-10 questionnaire scores and variables such as the WHO-FC and 6MWD, other clinical parameters were not included. Furthermore, the EmPHasis-10 questionnaire is a patient-reported outcome measure, making it inherently subjective and potentially influenced by patient comprehension, which may be contingent upon their educational level. Notably, our analysis did not yield evidence to suggest that educational attainment or environmental factors significantly affected these outcomes. The randomly selected time of patient inclusion (duration of the illness) in this analysis should also be considered.

In clinical practice, risk-assessment evaluation during follow-up should incorporate the patient’s perspective in addition to the currently used objective tools.

Our study’s limitations stem from being conducted at a single center and the small patient cohort, which mirrors the relatively low number of precapillary PH patients diagnosed nationwide. Furthermore, access to right heart catheterization for routine re-evaluation, as recommended by the 2022 ESC/ERS Guidelines, remains limited in Romania. These factors hindered our ability to include additional data that could have enhanced the robustness of our analysis.

Future research directions must focus on networking between all accredited Romanian PH centers to collect larger patient datasets. The influence of PH-specific drugs on the HRQoL and additional comprehensive echocardiographic parameters could be further studied.

## 5. Conclusions

This represents the first Romanian study to explore the correlations between two clinically applied assessment tools, the EmPHasis-10 questionnaire and the FSRA tool, and right heart echocardiographic parameters in patients with precapillary pulmonary hypertension, explicitly those with PAH and CTEPH, undergoing targeted medical treatment. Significant correlations were noted between the EmPHasis-10 score and WHO functional class, as well as the 6 min walking distance.

While the EmPHasis-10 score did not significantly correlate with echocardiographic measures, it was positively correlated with the FSRA tool. This study also identified significant correlations between the FSRA tool and echocardiographic parameters within the entire cohort, particularly with mPAP, TAPSE, RVOT-AT, and TAPSE/sPAP ratio.

FSRA-based assessment of 12-month mortality yielded results consistent with current clinical guidelines.

In conclusion, our study confirms an agreement between EmPHasis-10 scores and the FSRA tool, with poorer health-related quality of life corresponding to higher FSRA. The EmPHasis-10 questionnaire proves to be a valuable, easy-to-use instrument, offering meaningful insights into patients’ health-related quality of life and prognostic indices.

## Figures and Tables

**Table 1:** Baseline characteristics for the entire cohort and PAH and CTEPH groups.

Parameters	Entire Cohort	PAH Group	CTEPH Group	PAH Group vs. CPETH Group *p* Value
Patients (n, %)	40 (100)	27 (67.5)	13 (32.5)	-
Age (years; mean ± SD)	53.18 ± 18.56	48.78 ± 20.24	62.31 ± 9.91	0.006
Female (n, %)	23 (57.5)	16 (40)	7 (60)	-
BMI (kg/m^2^; median, minimum–maximum)	26.47 (15.63–46.84)	26.14 (15.63–41.76)	27.2 (18.9–46.84)	0.421
Urban (n, %)	22 (55)	16 (40)	6 (15)	-
Disabled/retired (n, %)	33 (82.5)	22 (55)	11 (27.5)	-
Level of education (n, %)				
1	3 (7.5)	2 (5)	1 (2.5)	-
2	5 (12.5)	3 (7.5)	2 (5)	-
3	24 (60)	16 (40)	8 (20)	-
4	3 (7.5)	2 (5)	1 (2.5)	-
5	0	0	0	-
6	5 (12.5)	4 (10)	1 (2.5)	-
WHO-FC (n, %)				
I	6 (15)	4 (10)	2 (5)	-
II	17 (42.5)	13 (32.5)	4 (10)	-
III	13 (32.5)	8 (20)	5 (12.5)	-
IV	4 (10)	2 (5)	2 (5)	-
6MWD (meters; mean ± SD)	366.4 ± 138.3	384.52 ± 124.86	352.69 ± 149.26	0.483
6MWD (n, %)				-
>440 m	13 (32.5)	10 (25)	3 (7.5)	-
320–440 m	15 (37.5)	11 (27.5)	4 (10)	-
165–319 m	9 (22.5)	4 (10)	5 (12.5)	-
<165 m	3 (7.5)	2 (5)	1 (2.5)	-
NT-proBNP (pg/mL; mean ± SD)	1914.88 ± 2569.28	1536.37 ± 2188.68	2704.9 ± 3201.55	0.163
NT-proBNP (n, %)				-
<300 pg/mL	13 (32.5)	10 (25)	3 (7.5)	-
300–649 pg/mL	6 (15)	3 (7.5)	3 (7.5)	-
650–1100 pg/mL	6 (15)	5 (12.5)	1 (2.5)	-
>1100 pg/mL	15 (37.5)	9 (22.5)	6 (15)	-
RHC parameters (median, minimum–maximum)				-
mPAP (mmHg)	45 (14–73)	50.5 (14–73)	41 (33–56)	0.100
PAWP (mmHg)	11 (3–54)	10.5 (3–54)	12 (4–22)	0.289
PVR (WU)	10 (1.59–28)	7.5 (1.59–28)	11.99 (3–21)	0.545
CO (L/min)	4.47 (1.62–9.31)	4.76 (2.13–9.31)	3.89 (1.62–8.3)	0.160
CI (L/min/m^2^)	2.5 (1.17–5.6))	2.82 (1.34–5.6)	2.3 (1.17–4.7)	0.194

6MWD, 6 min walking distance; BMI, body mass index; CI, cardiac index; CO, cardiac output; CTEPH, chronic thromboembolic pulmonary hypertension; mPAP, mean pulmonary arterial pressure; n, number of patients; NT-proBNP, N-terminal pro-B-type natriuretic peptide; PAH, pulmonary arterial hypertension; PAWP, pulmonary artery wedge pressure; PVR, pulmonary vascular resistance; RHC, right heart catheterization; SD, standard deviation; WHO-FC, World Health Organization functional class; WU, Wood units. Cardiac output was calculated using the Fick principle.

**Table 2:** The results of the EmPHasis-10 questionnaire for the entire cohort, PAH group, and CTEPH group, and stratification based on WHO-FC assessment.

Parameters	Entire Cohort	PAH Group	CTEPH Group	PAH Group vs. CTEPH Group *p* Value
EmPHasis-10 questionnaire (points; mean ± SD)	18.83 ± 12.71	20.11 ± 13.64	16.15 ± 10.53	0.499
EmPHasis-10 questionnaire (n, %)				-
0–16 points	19 (47.5)	13 (32.5)	6 (15)	
17–33 points	14 (35)	8 (20)	6 (15)	
34–50 points	7 (17.5)	6 (15)	1 (2.5)	
WHO-FC	EmPHasis-10 questionnaire (points; median, minimum–maximum)			
I	5.5 (0–14)	8.5 (3–14)	1.5 (0–3)	0.116
II	14 (2–36)	18 (4–36)	13 (2–34)	0.738
III	25 (7–46)	35 (7–46)	19 (11–29)	0.131
IV	22 (7–27)	15.5 (7–24)	23.5 (20–27)	0.475

CTEPH, chronic thromboembolic pulmonary hypertension; n, number of patients; PAH, pulmonary arterial hypertension; SD, standard deviation; WHO-FC, World Health Organization functional class.

**Table 3:** FSRA-based risk assessment for the entire cohort, as well as separately for the PAH and CTEPH groups, with results further stratified according to WHO functional class.

Parameters	Entire Cohort	PAH Group	CTEPH Group	PAH Group vs. CTEPH Group *p* Value
FSRA (points; mean ± SD)	2.25 ± 0.98	2.07 ± 0.87	2.54 ± 1.05	0.163
FSRA (n, %)				-
Low risk	10 (25)	7 (17.5)	3 (7.5)	
Intermediate–low risk	15 (37.5)	13 (32.5)	2 (5)	
Intermediate–high risk	11 (27.5)	4 (10)	7 (17.5)	
High risk	4 (10)	3 (7.5)	1 (2.5)	
WHO-FC	FSRA tool (points; median, minimum–maximum)			
I	1 (1–2)	1 (1–2)	1 (1–1)	0.541
II	2 (1–3)	2 (1–3)	2 (1–3)	0.543
III	3 (2–4)	3 (2–4)	3 (3–4)	0.444
IV	3 (2–4)	3 (2–4)	3.5 (3–4)	0.698

CTEPH, chronic thromboembolic pulmonary hypertension; FSRA, four-strata risk assessment; n, number of patients; PAH, pulmonary arterial hypertension; SD, standard deviation; WHO-FC, World Health Organization functional class.

**Table 4:** Echocardiographic assessment of right heart parameters in the entire cohort and in PAH and CTEPH groups.

Parameters	n	Entire Cohort (Mean ± SD)	PAH Group (Mean ± SD)	CTEPH Group (Mean ± SD)	PAH Group vs. CTEPH Group *p* Value
Peak TRV (m/s)	40	4.23 ± 0.69	4.26 ± 0.66	4.16 ± 0.74	0.644
TAPSE (mm)	40	18.71 ± 4.75	18.42 ± 4.39	19.3 ± 5.54	0.550
RVOT-AT (ms)	37	81.97 ± 14.45	81.83 ± 13.09	82.23 ± 17.26	0.938
TR pressure gradient (mmHg)	40	73.76 ± 22.79	74.61 ± 22.73	71.98 ± 23.73	0.737
IVC diameter (mm)	40	19.33 ± 4.46	19.33 ± 4.69	19.31 ± 4.13	0.987
sPAP (mmHg)	40	79.09 ± 23.22	80.43 ± 23	76.29 ± 24.34	0.604
TAPSE/sPAP ratio	40	0.26 ± 0.12	0.25 ± 0.1	0.28 ± 0.13	0.495
mPAP (mmHg)	37	39.18 ± 8.96	39.26 ± 8.11	39.01 ± 10.7	0.938

IVC, inferior vena cava; mPAP, mean pulmonary artery pressure; n, number of patients; RVOT-AT, right ventricular outflow tract acceleration time; SD, standard deviation; sPAP, systolic pulmonary artery pressure; TAPSE, tricuspid annular plane systolic excursion; TR, tricuspid regurgitation; TRV, tricuspid regurgitation velocity.

**Table 5:** The correlation of right ventricular echocardiographic parameters with EmPHasis-10 questionnaire scores and the FSRA tool.

Parameters	n	Correlation with EmPHasis-10 Questionnaire Score; r, *p* Value			Correlation with FSRA Tool r, *p* Value		
		Entire Cohort	PAH Group	CTEPH Group	Entire Cohort	PAH Group	CTEPH Group
Peak TRV (m/s)	40	0.16, 0.319	0.24, 0.226	−0.02, 0.943	0.13, 0.44	0.01, 0.977	0.42, 0.15
TAPSE (mm)	40	−0.19, 0.237	−0.12, 0.536	−0.35, 0.242	−0.46, 0.003	−0.527, 0.006	−0.56, 0.046
RVOT-AT (ms)	37	−0.03, 0.853	−0.11, 0.568	0.19, 0.544	−0.42, 0.01	−0.47, 0.013	−0.18, 0.552
TR pressure gradient (mmHg)	40	0.16, 0.334	0.24, 0.226	−0.03, 0.932	0.12, 0.45	0.01, 0.977	0.45, 0.124
IVC diameter (mm)	40	0.02, 0.881	−0.06, 0.779	0.3, 0.317	0.21, 0.195	0.23, 0.252	0.32, 0.281
sPAP (mmHg)	40	0.14, 0.381	0.23, 0.256	0.02, 0.945	0.15, 0.36	0.03, 0.891	0.49, 0.087
TAPSE/sPAP ratio	40	−0.21, 0.187	−0.18, 0.357	−0.11, 0.714	−0.43, 0.005	−0.25, 0.212	−0.305, 0.122
mPAP (mmHg)	37	0.03, 0.853	0.14, 0.505	−0.19, 0.544	0.42, 0.01	0.57, 0.004	0.18, 0.552

FSRA, four-strata risk assessment tool; IVC, inferior vena cava; mPAP, mean pulmonary artery pressure; n, number of patients; RVOT-AT, right ventricular outflow tract acceleration time; sPAP, systolic pulmonary artery pressure; TAPSE, tricuspid annular plane systolic excursion; TR, tricuspid regurgitation; TRV, tricuspid regurgitation velocity.

**Table 6:** Mortality rates—comparison between current ESC/ERS 2022 Guidelines and our research.

FSRA	Mortality Rates	
	2022 ESC/ERS Guidelines [1]	Current Research
Low risk	0–3%	0
Intermediate–low risk	2–7%	6.66% (1 case)
Intermediate–high risk	9–19%	9.09% (1 case)
High risk	>20%	50% (2 cases)

FSRA, four-strata risk assessment tool.

## Data Availability

The data presented in this study are available on request from the corresponding author in accordance with local and national regulations.
